# Preliminary assessment of the performance of oyster shells and chitin materials as adsorbents in the removal of saxitoxin in aqueous solutions

**DOI:** 10.1186/1752-153X-6-86

**Published:** 2012-08-14

**Authors:** Silvia P Melegari, William G Matias

**Affiliations:** 1 Laboratory of Environmental Toxicology (LABTOX), Department of Sanitary and Environmental Engineering, University Campus “Trindade”, Florianopolis, SC, CEP 88040-970, Brazil; 2 Laboratório de Toxicologia Ambiental, LABTOX, Depto. de Engenharia Sanitária e Ambiental, Universidade Federal de Santa Catarina, Campus Universitário, CEP: 88040-970, Florianópolis, SC, Brasil

**Keywords:** Saxitoxin, Chitin, Oyster shell, Adsorption isotherm, Adsorption kinetic

## Abstract

**Background:**

This study evaluated the adsorption capacity of the natural materials chitin and oyster shell powder (OSP) in the removal of saxitoxin (STX) from water. Simplified reactors of adsorption were prepared containing 200 mg of adsorbents and known concentrations of STX in solutions with pH 5.0 or 7.0, and these solutions were incubated at 25°C with an orbital shaker at 200 RPM. The adsorption isotherms were evaluated within 48 hours, with the results indicating a decrease in STX concentrations in different solutions (2–16 μg/L). The kinetics of adsorption was evaluated at different contact times (0–4320 min) with a decrease in STX concentrations (initial concentration of 10 μg/L). The sampling fractions were filtered through a membrane (0.20 μm) and analyzed with high performance liquid chromatography to quantify the STX concentration remaining in solution.

**Results:**

Chitin and OSP were found to be efficient adsorbents with a high capacity to remove STX from aqueous solutions within the concentration limits evaluated (> 50% over 18 h). The rate of STX removal for both adsorbents decreased with contact time, which was likely due to the saturation of the adsorbing sites and suggested that the adsorption occurred through ion exchange mechanisms. Our results also indicated that the adsorption equilibrium was influenced by pH and was not favored under acidic conditions.

**Conclusions:**

The results of this study demonstrate the possibility of using these two materials in the treatment of drinking water contaminated with STX. The characteristics of chitin and OSP were consistent with the classical adsorption models of linear and Freundlich isotherms. Kinetic and thermodynamic evaluations revealed that the adsorption process was spontaneous (ΔG_ads_ < 0) and favorable and followed pseudo-second-order kinetics.

## Background

The blooming of cyanobacteria in drinking water reservoirs is an increasingly common problem associated with eutrophication, and several different water treatment processes exist to remove cyanobacteria and cyanotoxins [1]. When cyanobacteria lyse due to natural causes or through the use of algaecides, cyanotoxins are released and solubilized in water [1,2]. When this occurs, the water treatment process must ensure the efficient and consistent removal of cyanotoxins. Appropriate treatment processes exist and have been tested and optimized to remove soluble organic compounds. Such processes include using ozone, activated charcoal, nanofiltration or reverse osmosis, and biodegradation [1-6].

Consistent evidence indicates that high doses of powdered activated charcoal work well in the removal of cyanotoxins from aqueous solutions, but this process is slow and expensive due to the large quantities of activated charcoal that must be used [7,8]. Different alternatives for removing cyanotoxins by adsorption and with others techniques have been tested [9-12], but the evidence regarding the usefulness of filters made from natural materials to remove cyanotoxins, in particular saxitoxin (STX), is still unreliable. Another technology employed in recent years to remove cyanotoxins from aqueous solutions is carbon nanotubes, which have a high adsorption capacity compared to activated charcoal and other conventional adsorbents, such as mineral clays [13], but this technology has not been comprehensively tested for its toxicology effects. Saxitoxin (STX) is a neurotoxin classified as belonging to the paralytic shellfish poisoning (PSP) toxins and has been identified for the first time in freshwater contaminated by *Aphanizomenon flos-aquae*[14]. Apart from this species, STX can be synthesized by other species of cyanobacteria such as *Cylindrospermopsis raciborskiiLyngbya* and *Anabaena*[15,16]. The rapid action of STX blocking sodium channels in nerve axons and causing loss of sensation and paralysis results in highly neurotoxic and potentially lethal effects 2–25 hours after ingestion. The LD50 in mice is of 8–10 μg/kg i.p., 3.4 μg/kg g i.v. and 260 μg/kg by the oral route [17]. Data regarding the cytotoxic and genotoxic effects of STX are very scarce, however its toxicity *in vitro* has been reported by Perreault et al. [18] and Melegari et al. [19]. Guidelines for cyanobacterial toxins in water, including PSP, exist in several countries worldwide and have usually arisen as a consequence of cyanobacterial contamination in drinking water reservoirs [12]. Water treatment systems can eliminate cyanobacteria and their toxins from raw water, but conventional water treatment has proven ineffective at removing dissolved cyanotoxins from water [12,20].

The shells of crustaceans and mollusks are an abundant waste product of the fishing industry, which often considers them pollutants. Recycling the shells can reduce their environmental impact on the locations where such waste is generated and stored [21]. Reprocessing these materials has become very important from an environmental and economic perspective because it can eliminate waste in the fishing industry and can reduce the final cost of crustacean and mollusk acquisition by approximately 60% [22]. These materials have generally been used in studies on the adsorption of heavy metals such as Cu (ІІ), Ni (II) Zn (ІІ), Cr (VI), Cd (II) and Pb (II) from various aqueous solutions [23-27].

The shells of shellfish are primarily composed of aragonite, which is a mineral modification of calcium carbonate. Calcium carbonate (CaCO_3_) is commonly found in one of three mineral modifications. The first polymorph, calcite, is the most common mineral modified from CaCO_3_ and is the main constituent of vast sedimentary limestone rock formations [28]. The occurrence of aragonite, the second polymorph, is linked to certain physical and chemical conditions during its formation. The third polymorph, vaterite, is a much scarcer mineral [28]. Aragonite has a more compact atomic arrangement than calcite and is found in shells, pearls and coral. The use of this biogenic calcium carbonate has greatly contributed to the removal of phosphates [29,30] and heavy metals [23,24] from water and offers a greater active surface and more adsorbent sites than calcite. The adsorption capacity of aragonite, based on the number of moles of phosphate adsorbed per gram of particle, is approximately 20 times greater than that of calcite [31].

Chitin is a linear chain polysaccharide composed of units of N-acetyl-2-dioxin-D-glucopyranoside linked by glycosidic β (1 → 4) bonds. Chitin is a renewable material from natural sources and is biodegradable, nontoxic and insoluble in water and many organic solvents. The primary source of chitin for use in laboratories is the exoskeletons of various crustaceans such as crab and shrimp [32-37]. Chitin has been strongly associated with proteins, inorganic compounds, pigments and lipids [32,33], and several methods have been employed to remove these impurities; however, no standard purification process exists currently. Deproteinization, demineralization and depigmentation via digestion with strong alkalis and acids has been required to isolate chitin [36].

This study evaluated the adsorption capacities of the natural materials chitin and oyster shell powder (OSP) in removing STX from aqueous solutions and assessed their potential for use as alternative, low-cost and non-toxic adsorption materials. The materials were tested at different pH levels, 5.0 and 7.0, to provide the water industry with guidance on their use for the removal of STX.

## Results and discussion

### Effects of STX concentration and contact time

**Figure 1:**
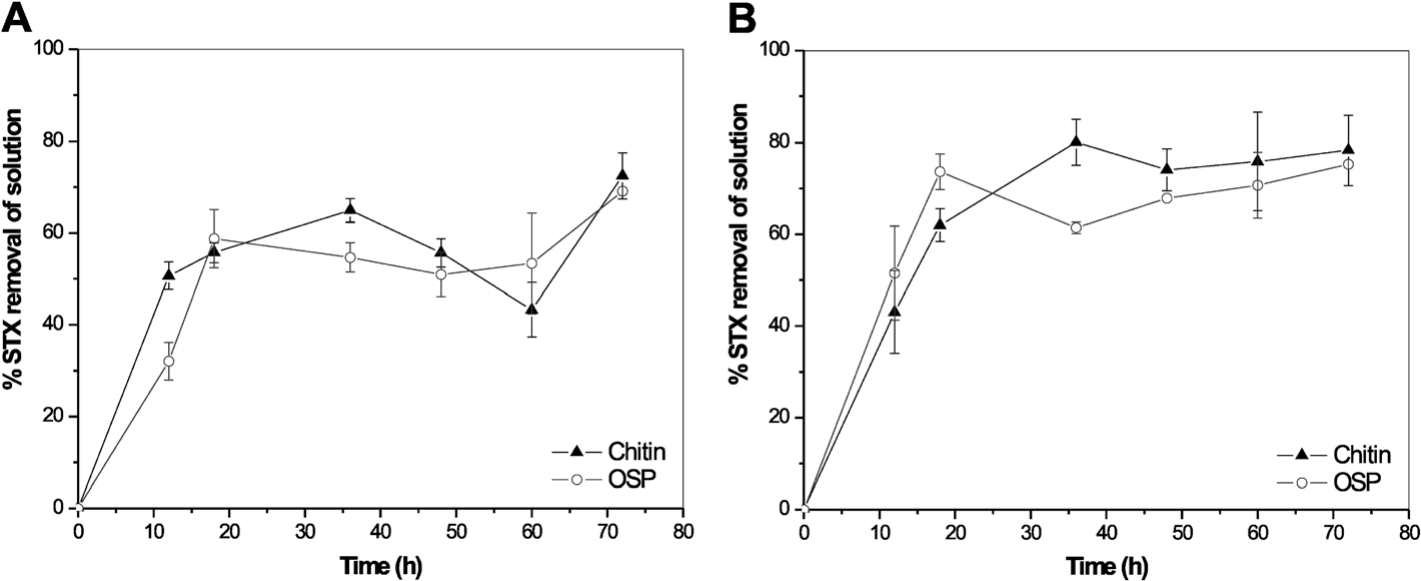
Removal process of STX from aqueous solutions using adsorption onto chitin and OSP at different contact times and at a constant temperature (25°C) in solutions with a pH of (A) 5.0 and (B) 7.0.

STX was removed from aqueous solution when it came into contact with the adsorbents being tested (Figure 1), and the kinetics of its adsorption, with respect to contact time at each pH used for chitin and OSP, indicated significant removal of STX by both materials (≥ 50% removal) when tested for 18 h of contact time. No change in toxin concentration was observed during the contact time in the control experiment, in which STX was present without an adsorbent. These results demonstrated that the adsorption mechanisms occurred in the presence of the adsorbents evaluated (chitin and OSP) and that no degradation of the STX existed over the contact time of the experiment.

The removal rate of STX by the adsorbents decreased with an increase in contact time, likely due to saturation of the adsorbing sites. Such behavior suggested that adsorption occurred through ion exchange mechanisms in this case. Shi et al. (2012) has reported that neutrally charged STX predominates between pH 9 and 12 and is the species that would be most likely to have the highest adsorption by activated carbons based on its non-electrostatic interactions. The pH range employed for our tests (5 and 7) most likely induced electrostatic interactions because of the amine groups in the STX structure that had the potential to gain protons, depending on the pH of the solution. However, this chemical interaction between adsorbent and adsorbate requires further investigation.

The saturation of the adsorbent sites may also have been associated with the presence of a phosphate buffer solution to control the medium pH, especially in the case of OSP. Ion exchange between the ions of the buffer solution and adsorbents tests has been reported by other authors [9,10]. The presence of the buffer solution in the adsorption tests was important to ensure a stable pH, given that the presence of these adsorbents in aqueous solution, especially OSP [23], has been found to significantly alter a pH medium to ≥ 6.0. During the experiment using a solution of pH 7.0, following 48 h of contact time, the remaining concentration of STX in aqueous solution tended to stabilize, most likely because it reached its adsorption capacity and attained equilibrium between the adsorbents and adsorbate. Adsorption equilibrium did not occur for either adsorbent when the solution had a pH of 5.0, even after 72 h of contact time. In this case, the pH seemed to interfere with the achievement of adsorption equilibrium because both adsorbents experienced a delay in reaching an equilibrium at pH 5.0, indicating non-specific adsorption reactions and a desorption mechanism [11]. The loss of adsorbent weight by solubilization mechanisms in aqueous media can occur under acidic conditions. For example, CaCO_3_, the primary constituent of OPS, is more soluble under these conditions.

### Considerations regarding adsorption isotherms

The data obtained at equilibrium (t = 4320 min) were evaluated in three different isotherm models that described which adsorption mechanism occurred in each experiment: the linear model (Eq. 1), the Langmuir model (Eq. 2) and the Freundlich model (Eq. 3) [9-11,24]. The Freundlich model has recently been widely used to determine the adsorption of cyanotoxins in sediment [9-11]. The mass (μg) of STX adsorbed per kilogram of adsorbent was quantified from the concentration of STX remaining in aqueous solution, and the adsorption constants were calculated from the adsorption isotherms [9-11,38].

(1) $$Q_{\text{e}}=K_{\text{d}}C_{\text{e}}$$

(2) $$\frac{C_{\text{e}}}{Q_{\text{e}}}=\frac{1}{K_{\text{ads}}Q_{\text{m}}}+\frac{C_{\text{e}}}{Q_{\text{m}}}$$

(3) $$\text{log}Q_{\text{e}}=\text{log}K_{\text{F}}+\frac{1}{\text{n}}\text{log}C_{\text{e}}$$

where Q_e_ is the amount of adsorbed STX (μg/kg), C_e_ is the STX equilibrium concentration of the adsorbate (μg/L), K_d_ is the linear distribution constant (μg/kg), Q_m_ is the maximum adsorption capacity (μg/kg), K_ads_ is the constant of equilibrium adsorption, and K_F_ and n are Freundlich constants related to the adsorption capacity (μg/kg) and adsorption intensity, respectively. A straight line with a slope of K_d_ was obtained in the linear model when Q_e_ was plotted against C_e_. When C_e_/Q_e_ was plotted against 1/Q_m_ in the Langmuir model, a straight line with a slope of 1/K_d_ and an intercept of C_e_/Q_m_ was obtained. Finally, a straight line with a slope of 1/n and an intercept at log K_F_ was obtained in the Freundlich model when logQ_e_ was plotted against log C_e_.

**Figure 2:**
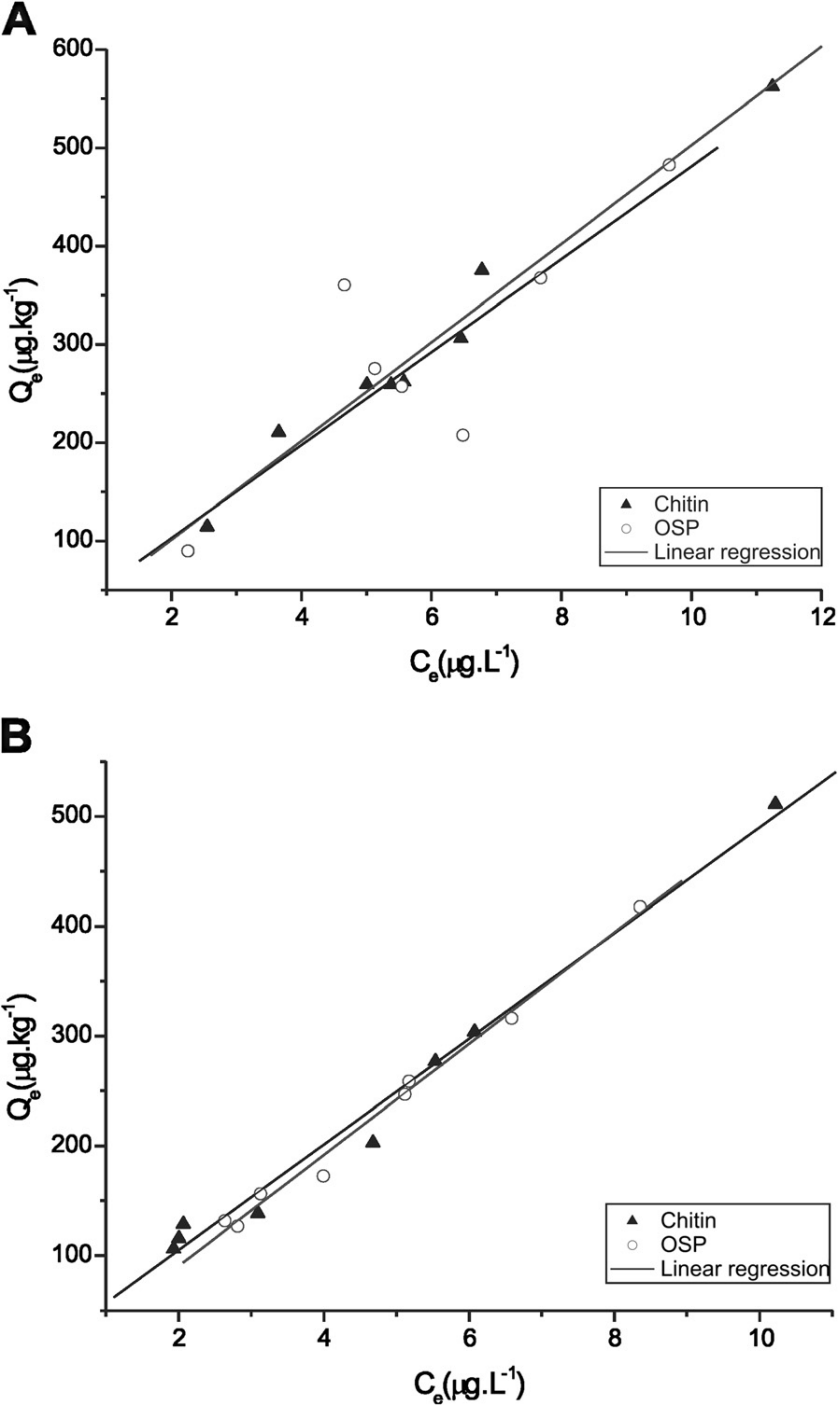
Linear isotherms for the adsorption of STX onto chitin and OSP at 25°C with a pH of (A) 5.0 (p <0.05) and (B) 7.0 (p <0.05).

**Figure 3:**
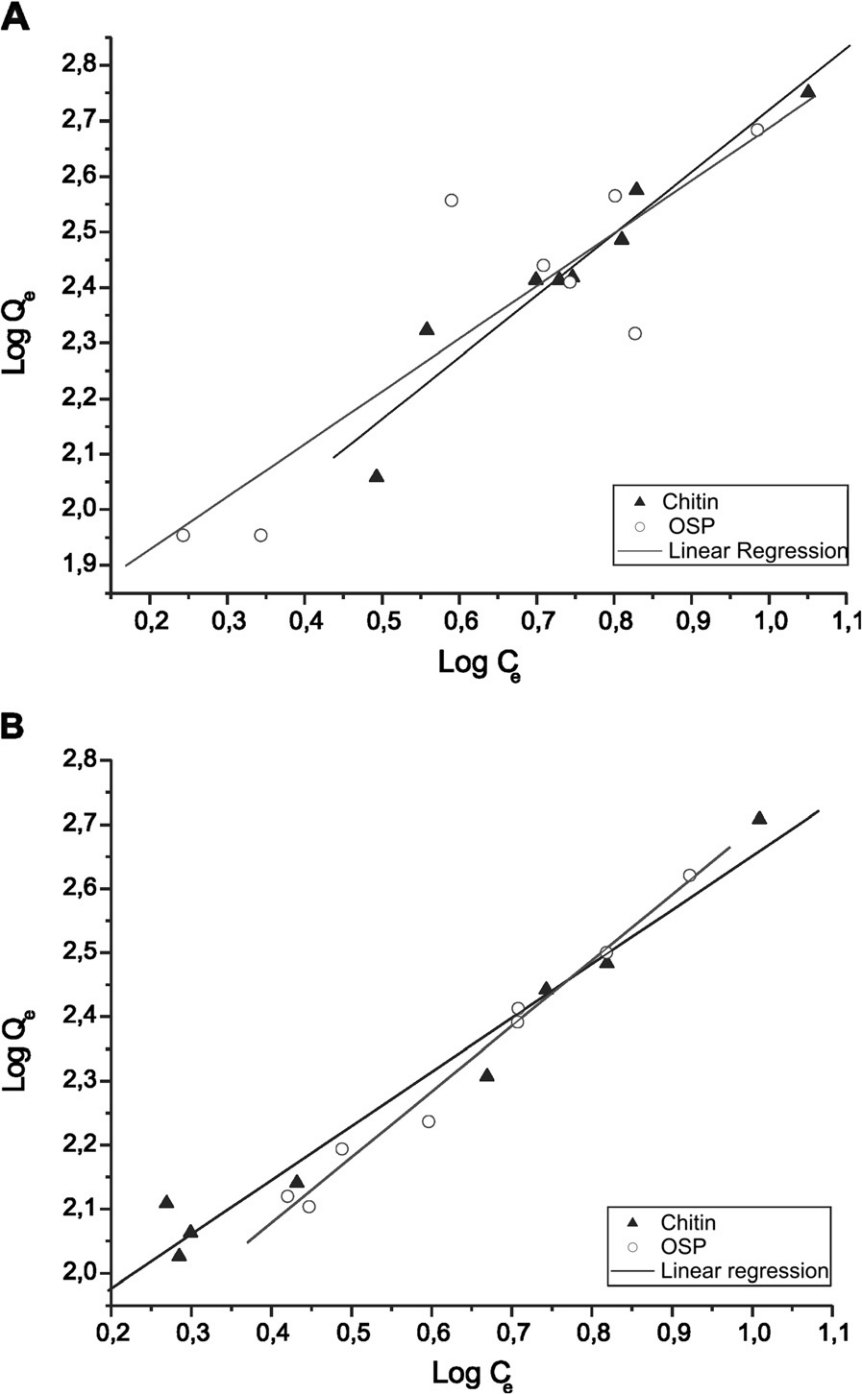
Freundlich isotherms for the adsorption of STX onto chitin and OPS at 25°C with a pH of (A) 5.0 (p <0.05) and (B) 7.0 (p <0.05).

The linear equations were obtained from the adsorption isotherms of the STX and were properly adjusted to the linear model (Figure 2) and the Freundlich model (Figure 3) of the adsorbents when tested at 25°C. The values of K_d_, K_F_ and n are presented in Table 1. These data did not fit within the acceptable linearity of the Langmuir model (R^2^ < 0.7; data not shown), suggesting that the surface of the adsorbent particles was not homogeneous and that the adsorption sites did not have equivalent adsorbent energy; this would be equivalent to a monolayer adsorption and consistent with a chemisorption process described by this model [39].These results suggest the occurrence of relatively weak intermolecular attraction between the adsorbate and absorbents. 

**Table 1:** Linear and Freundlich constants for the adsorption of STX onto chitin and OSP

**Adsorbents**	**pH**	**Linear**		**Freundlich**		
		***K***_***d***_***(μg/kg)***	***R***^***2***^	***n***	***K***_***F***_***(μg/kg)***	***R***^***2***^
Chitin	5.0	50.17	0.987	0.90	40.46	0.958
	7.0	48.14	0.992	1.18	64.24	0.983
OSP	5.0	47.31	0.873	1.05	54.70	0.869
	7.0	50.65	0.995	0.98	46.65	0.992

When using chitin as an adsorbent, both pH values showed an acceptable fit with the linear and Freundlich models: their R^2^ values ranged from 0.958 to 0.992. The high values of K_d_ (48.14-50.17 μg/kg) and K_F_ (40.46-64.24 μg/kg) indicated a high chemical affinity of this adsorbent with STX. The n values ranged from 0.90-1.18 and indicated that the adsorption of STX onto chitin could be considered a favorable process, which confirmed the heterogeneity and appropriate distribution of the adsorption energy sites.

When using OSP as an adsorbent, both linear and Freundlich models indicated an acceptable fit at a pH of 7.0, where the R^2^ values ranged from 0.992-0.995. Under conditions with a pH of 5.0, the R^2^ values ranged from 0.869-0.873. In this case, the fit to the models was influenced by the relationship between the pH medium and the composition of the adsorbent. OSP is composed mostly of approximately 60% calcium carbonate [24], which has increased solubility in acidic conditions. High values of K_d_ (47.31 to 50.65 μg/kg) and K_F_ (46.6 to 52.9 μg/kg) reflected a high affinity of the OSP to the STX. The n values ranged from 0.98-1.05 and indicated that the adsorption of STX onto OSP could also be considered favorable, especially at a pH of 7.0.

According to the Freundlich adsorption isotherm, the fraction of surface coverage (Q_e_) is related to the equilibrium concentration of the adsorbate. The Freundlich isotherm describes equilibrium on heterogeneous surfaces and assumes a monolayer adsorption capacity [40]. This explanation could justify the good fits of the materials used in this model because these adsorbents came from natural sources and were expected to exhibit the behavior of heterogeneous surfaces. A pH of 7.0 demonstrated more favorable adsorption (R^2^ = 0.983-0.995) from both adsorbents tested, which was justified by the behavior observed in the kinetics of adsorption, where the adsorption conditions reached equilibrium in 48 h, but such behavior was not observed at a pH of 5.0.

### Considerations regarding the kinetics of adsorption

The kinetics of adsorption were analyzed using the Lagergren pseudo-first-order (Eq. 4) and pseudo-second-order (Eq. 5) equations [24]:

(4) $$\text{In}\left(Q_{\text{e}}-Q_{\text{t}}\right)=\text{In}\;Q_{\text{e}}-{\text{k}}_{1}\;\text{t}$$

(5) $$\frac{\text{t}}{Q_{\text{t}}}=\left[\frac{1}{k_{2}{Q_{\text{e}}}^{2}}\right]+\left(\frac{1}{Q_{\text{e}}}\right)\text{t}$$

where Q_t_ is the amount of adsorbed STX per kilogram of adsorbent material (μg/kg) over time t, Q_e_ is the amount of adsorbed STX per kg of adsorbent material in equilibrium, k_1_ is the constant (1/min) of the pseudo-first-order model, and k_2_ is the constant (kg/μg min) of the pseudo-second-order model. The product k_2_Q_e_^2^ is the initial adsorption rate in the pseudo-second-order model, called *h*, the values of which (μg/kg min) are presented in Table 2. 

**Table 2:** Kinetic parameters for the adsorption of STX onto chitin and OSP under pH conditions of 5.0 and 7.0, at 25 °C

**Adsorbents**	**pH**	**Pseudo-first-order**		**Pseudo-second-order**			
		***k***_***1***_***(1/min)***	***R***^***2***^	***Q***_***e***_***(μg/kg)***	***h (μg/kg min)***	***k***_***2***_***(kg/mg min)***	***R***^***2***^
Chitin	5.0	3.55x10^-4^	0.7015	326.80	3.96 x10^4^	0.25x10^-6^	0.9734
	7.0	10.2x10^-4^	0.8830	500.00	49.4 x10^4^	2.03x10^-6^	0.9433
OSP	5.0	3.22x10^-4^	0.8700	370.37	25.8x10^4^	2.22x10^-6^	0.9428
	7.0	6.96x10^-4^	0.8805	400.00	26.8 x10^4^	3.72x10^-6^	0.9015

**Figure 4:**
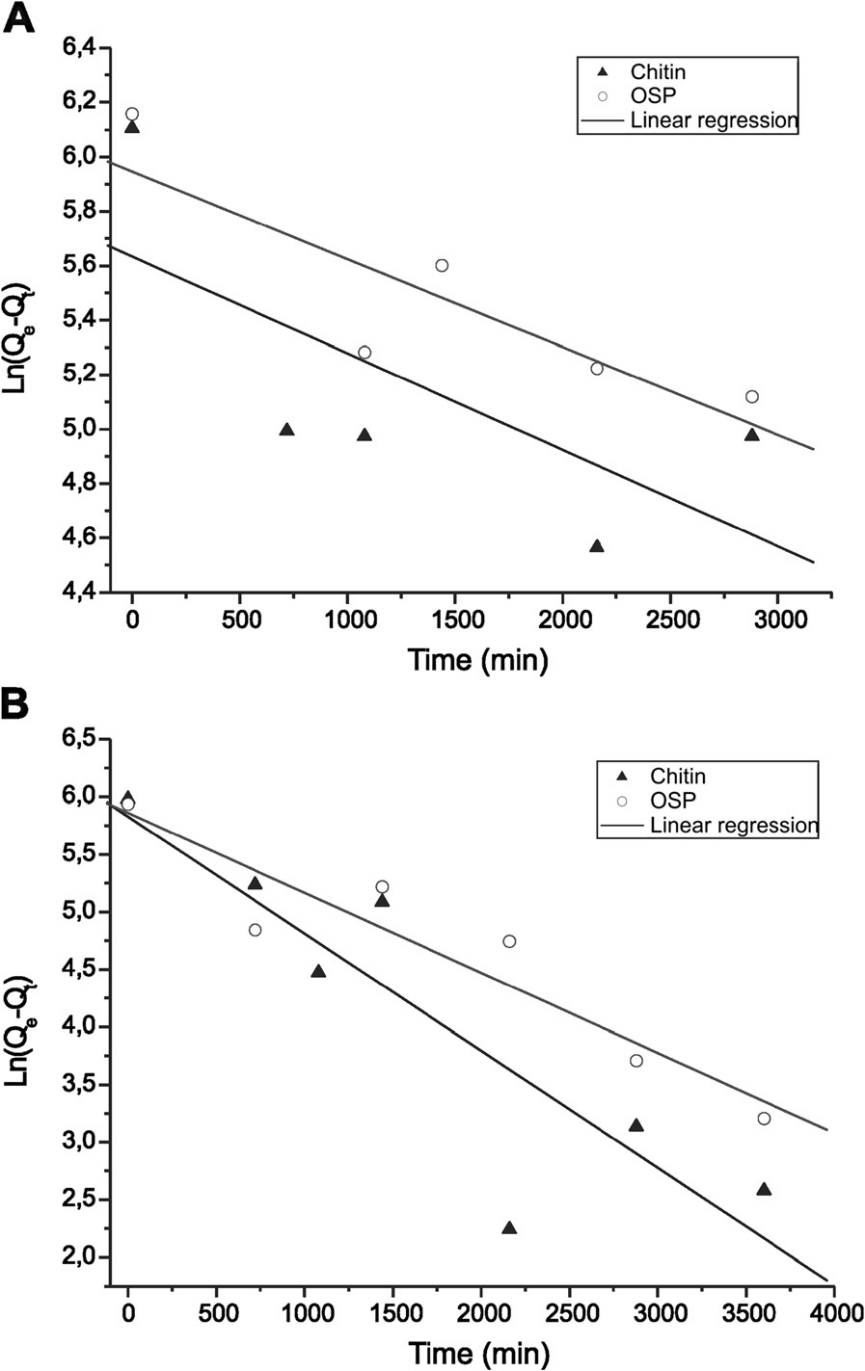
Plots of the pseudo-first-order kinetics for the adsorption of STX with a pH of (A) 5.0 (p <0.05) and (B) 7.0 (p <0.05) onto chitin and OSP at 25°C.

**Figure 5:**
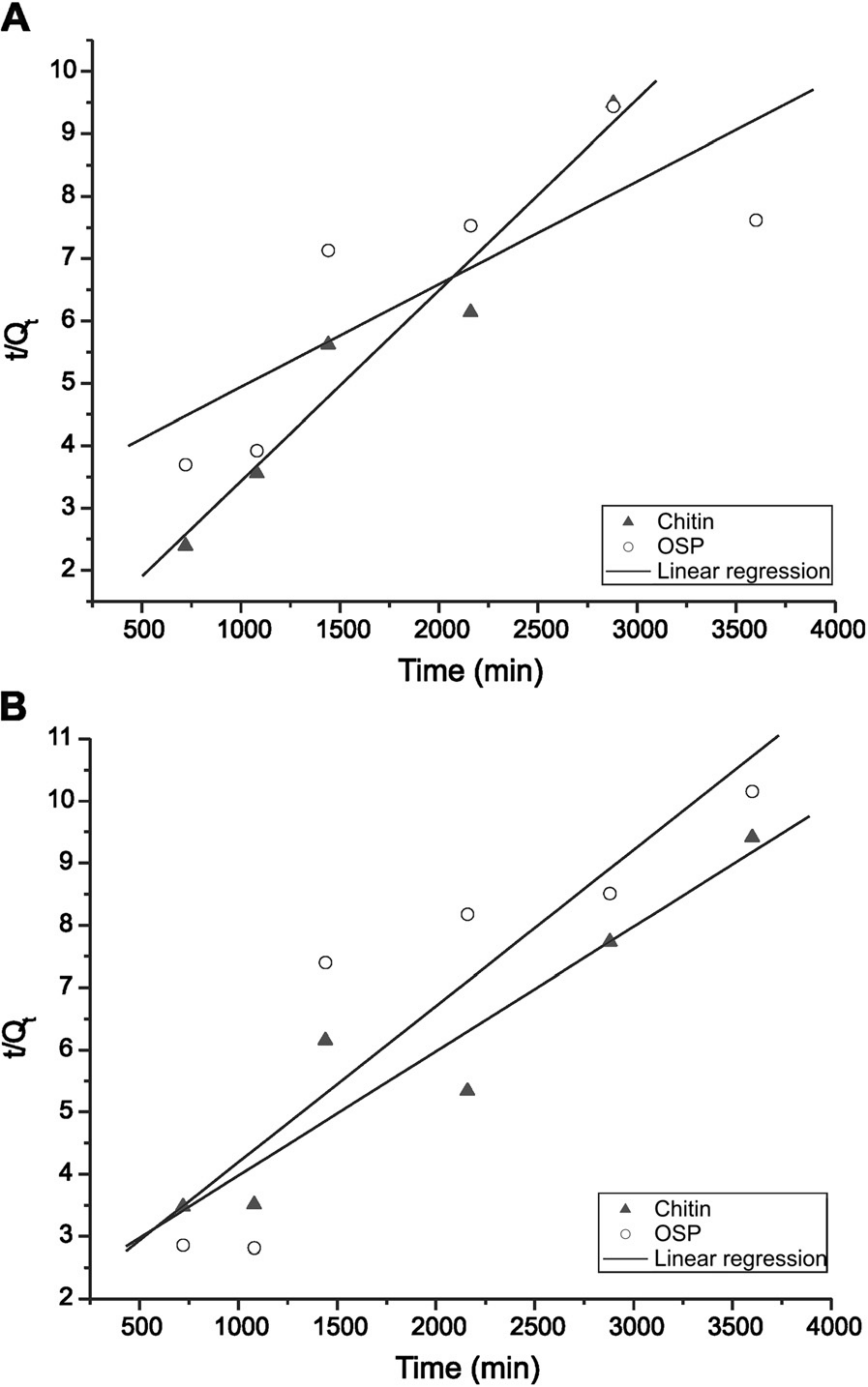
Plots of the pseudo-second-order kinetics for the adsorption of STX with a pH of (A) 5.0 (p <0.05) and (B) 7.0 (p <0.05) onto chitin and OSP at 25°C.

Figure 4 and Figure 5 show the kinetics of adsorption of STX onto chitin and OSP and are adjusted to the kinetic models of pseudo-first-order and pseudo-second-order, respectively. The parameters obtained from these analyses are listed in Table 2. The pseudo-first-order model did not indicate an adjusted fit for the chitin and OSP (R^2^ = 0.7010-0.8805) and did not represent the kinetics of adsorption as well as the pseudo-second-order model, which had better linearity and confirmed the nature of second-order process. These results confirm the results of Hsu [24] for OSP, who had tested the adsorption of metals. The maximum initial sorption rate observed at a pH of 7.0 was 49.3x10^4^ μg/kg min for chitin, and 26.8x10^4^ μg/kg min for OSP. The maximum initial sorption rate of both adsorbents was greater at a pH of 7.0 than at 5.0. 

### Thermodynamic considerations

The Gibbs free energy of adsorption (ΔG_ads_) can be calculated from the isotherm adsorption, as reported by several authors [24,26] according to Equation (6):

(6) $$\Delta {\text{G}}_{\text{ads}}=-2.303\;\text{R}\;\text{T}\;\text{logK}$$

where R is the universal gas constant, T is temperature and K is the adsorption constant. Using the values of K_F_ calculated by the Freundlich isotherm, the values of ΔG_ads_ (kJ/mol) to STX were calculated for the adsorbents tested at both pH levels (Table 3). 

**Table 3:** Thermodynamic parameters for the adsorption of STX onto chitin and OSP

**Adsorbents**	**pH**	***ΔG***_***ads***_***(kJ/mol)***
Chitin	5.0	−9.182
	7.0	−10.324
OSP	5.0	−9.843
	7.0	−9.530

The values of ΔG_ads_ < 0 indicated that the adsorption of STX onto chitin and OSP was spontaneous and favorable in experimental conditions, and also showed that the mechanism of chemical adsorption was applicable to removing STX when using both adsorbents. These values could be further improved by favoring a more effective adsorption using combinations of efficient adsorbents for the adsorption of STX, such as clays and/or nanomaterials [9,13] or increasing the process temperature [24].

Chitin and OSP, both considered waste materials by the fishing industry, showed potential for use as adsorbents in the removal of STX from aqueous solutions under the experimental conditions in this study. The adsorbents show high adsorption capacity, besides offer significant advantages over currently expensive commercially available (e.g., activated carbons) [41]. However, further investigation should be conducted to explain the interaction between the adsorbents and adsorbate, optimize the pH conditions, perform recovery studies on both adsorbate and adsorbent, and research pilot-plant studies to assess the best contact time and rate of saturation for these adsorbents.

## Conclusions

This study found that chitin and OSP have the capacity to remove STX from aqueous solutions (> 50% in 18 h) at the concentrations studied. Freshwater is susceptible to contamination by STX, and both of the studied adsorbents proved to be viable alternatives for use in drinking water treatment. The rate of STX removal by the adsorbents progressively decreased with contact time, possibly due to saturation of the adsorbing sites, suggesting that the adsorption occurred via ion exchange mechanisms. The adsorption equilibrium was influenced by the pH of the reaction medium and was unfavorable under acidic conditions. The chitin and OSP characteristics were consistent with the classical linear and Freundlich isotherm models of adsorption, with the measured adsorption coefficients ranging from 40.46-64.24 μg/kg for chitin and 46.65-54.70 μg/kg for OSP. Thermodynamic and kinetic evaluations revealed that the adsorption process was spontaneous (ΔG_ads_ < 0) under favorable kinetics and tended to follow a pseudo-second-order process (R^2^ = 0.9015-0.9734). However, additional studies must be conducted to evaluate the viability of using of these materials on a real-world scale and could include experiments employing pilot-plant studies to assess the best contact time and rate of saturation of these adsorbents.

## Methods

### Adsorbents and simplified reactor of adsorption

Chitin was produced from shrimp shells and was provided by the Research Group on Chitin and Technological Applications (QUITECH) at the Federal University of Santa Catarina, Brazil, under the direction of Dr. Mauro Cesar Marghetti Laranjeira. Crude chitin was triturated and sieved in an 18-mesh sieve (particle size ≤ 1.0 mm). OSP (*MundoVerde*®) was produced from calcined oyster shells (650°C) and triturated to a particle size of ≤ 1.0 mm. The major component of the OSP at calcinations temperature has been found to be CaCO_3_ according to Choi et al. [42].

The simplified reactors of adsorption in this experiment were structured on a closed model and consisted of 50 mL cylindrical centrifuge tubes with hermetic covers that were adapted to the conditions of the assay. An orbital shaking incubator (CIENTEC, model CT-712r) was used to control temperature and agitation. The adsorbent materials, which had previously been dried at 105°C for 24 hours, were weighed on reactors, and the adsorption systems were sterilized in an autoclave for 15 minutes at 121°C to eliminate interference by the degradation of STX via different pathways.

### Adsorption assays

Adsorption kinetics were analyzed using 200 mg of adsorbent and 10 mL of a solution of STX (10 μg/L) prepared at either pH 5.0 or pH 7.0 and at 25°C under agitation (Shaker) at 200 rpm. The mixing times were evaluated in triplicate at an interval of 0 – 72 h. Aliquots of 500 μL were collected every 12 h and filtered through 0.20 μm membranes by centrifugation at 2000 g for 5 min and collected in microcentrifuge tubes.

The method used to evaluate the adsorption process was based on the studies of Miller et al. [10] and Ohe [43]. 10 mL of solution containing STX (2–16 μg/L in triplicate) at pH 5.0 or 7.0 were added to the adsorbent (200 ± 5 mg). The simplified reactors were shaken at 200 rpm for 48 hours at 25°C. Aliquots of 500 μL were filtered through 0.20 μm membranes by centrifugation at 2000 g for 5 min and collected in microcentrifuge tubes. The fractions were further analyzed with high performance liquid chromatography (HPLC) for the quantification of STX.

A control group was evaluated under the same conditions as the samples in both tests, but did not include the presence of adsorbents. The control group contained only the STX solutions at pH of 5.0 or 7.0, with the respective concentrations in sterile simplified reactors. This control was verified to assess the effect of toxin degradation by chemical, enzymatic or microbiological pathways.

### Analysis of STX

The adsorbate STX was obtained as a certified standard from the Institute for Marine Biosciences (Halifax, NS, Canada). The derivatization and quantification of the STX was adapted from the methodology recommended by the Association of Official Analytical Chemists (AOAC) [44]. The STX was derivatized by peroxide oxidation with a pre-column method. Briefly, 25 μL of an aqueous solution of H_2_O_2_ 10% (v:v) was added to 250 μL of 1 M NaOH in a microcentrifuge tube and mixed by vortexing. This solution was then added to 100 μL of a solution containing STX, mixed and allowed to react for 2 min at room temperature. Following the two-minute reaction time, the solution was added to 20 μL of concentrated acetic acid and mixed, yielding derivatized STX that could be quantified. The derivatized STX was quantified with an HPLC method using external calibration. The standard and derivatized samples (50 μL each) were injected into a Supelco Discovery® C18 column (250x4.0 mm, 5 mm i.d.). The HPLC operated under solvent gradient conditions using a buffered solution of ammonium formiate (0.1 M, pH 6.0) for eluent A and HPLC grade acetonitrile for eluent B. The chromatographic run duration was 15 minutes, and the gradient consisted of 0-1% eluent B during the first 5 minutes, 1-4% eluent B over the next 3 minutes, 4% eluent B for the following 5 minutes, 4-0% eluent B in the final 2 minutes, and a post-run of 3 minutes to stabilize the system before the next injection. The flow rate was 1 mL/min, and the method of detection was fluorescence spectrophotometry with an excitation wavelength of 340 nm and emission wavelength of 390 nm.

### Statistical analysis

All results were expressed as the mean ± standard deviation of the triplicates. Regression analysis, including a correlation factor (R^2^) and line equation, was performed using Origin® software (Northampton, MA-USA), and a p-value of <0.05 was accepted for the adsorption isotherm and kinetics of adsorption data.

## Competing interests

The authors declare that they have no direct financial relationships with the commercial identities MundoVerde®, Origin® and Discovery®, and they have no competing interests with these identities.
